# The Prevalence of Anxiety and Depressive Symptoms Among Patients With Celiac Disease in Jordan

**DOI:** 10.7759/cureus.39842

**Published:** 2023-06-01

**Authors:** Sara Haj Ali, Rahaf Alqurneh, Awni Abu Sneineh, Bandar Ghazal, Lana Agraib, Layali Abbasi, Sufian Rifaei, Tarek Mazzawi

**Affiliations:** 1 Medicine, Al-Balqa Applied University, Al-Salt, JOR; 2 Gastroenterology and Hepatology, University of Jordan, Amman, JOR; 3 Food Science and Nutrition, Jerash University, Jerash, JOR

**Keywords:** gluten-free diet, psychiatric disorder, depression, anxiety, celiac disease

## Abstract

Background

Celiac disease is an immune-mediated intestinal disorder with a global prevalence of 1% that results from gluten sensitivity in a genetically susceptible person. It presents with gastrointestinal symptoms, consequences of malabsorption, and/or extraintestinal manifestations that include neuropsychiatric symptoms.

Aim

The aim of this study was to measure the frequency of anxiety and depressive symptoms in Jordanian patients with celiac disease.

Methods

This was a cross-sectional study. A questionnaire was sent electronically to celiac disease patients who were members of the Friends of Celiac Disease Patients Association through WhatsApp using Google Forms (Google, Mountain View, California). The questionnaire contained demographic and disease-related questions, in addition to questions that assessed anxiety and depressive symptoms using validated Arabic versions of the Generalized Anxiety Disorder-7 score and Patient Health Questionnaire-9, respectively.

Results

A total of 133 patients answered the questionnaires. Of the respondents, 82.7% were females, and the mean age was 33.9 +/- 11.22 years; 31.6% of patients were non-compliant with a gluten-free diet, and 56.4% were symptomatic at the time of the questionnaire. The prevalence of anxiety and depressive symptoms were 85% and 82.7%, respectively. There was no correlation between any of the variables and the presence of anxiety or depressive symptoms.

Conclusion

A significant proportion of celiac disease patients in Jordan have evidence of anxiety and depressive symptoms. Given this high prevalence and the possible impact on the quality of life, physicians need to screen patients for the presence of psychiatric comorbidities and refer those who have symptoms for further evaluation.

## Introduction

Celiac disease (CD) is a chronic autoimmune enteropathy that leads to small intestinal mucosal changes resulting in malabsorption. It is triggered by the consumption of gluten in a person who is genetically predisposed [1]. Globally, the prevalence of positive celiac antibodies is 1.4%, while that of histologically-confirmed celiac is 0.7%, the highest prevalence being reported in Northwest Europe and the lowest in South America [2]. A previous Jordanian study showed that the incidence of celiac disease was 1:2800 live births with a prevalence of 7:100000 [3].

Classically, it was considered a disease of infants. However, it can develop at any age, and multiple studies reported a shift in the age of diagnosis in children with a median age of eight years [4-6].

Clinical presentation in adults includes gastrointestinal symptoms like diarrhea and flatulence, along with consequences of malabsorption that include weight loss, anemia or iron deficiency, and osteopenia. Extraintestinal manifestations include dermatitis herpetiformis, elevated liver enzymes, infertility, and neuropsychiatric manifestations.

The primary treatment of celiac disease is a life-long gluten-free diet (GFD) and repletion of nutritional deficiencies [7].

Previous studies reported an association between celiac disease and psychiatric disorders [8-11]. A meta-analysis and systematic review by Clappison et al. found a significant increase in the risk of eating disorders, anxiety, depression, autism, and attention deficit hyperactivity disorder [12]. The prevalence rate of depressive symptoms in celiac disease patients ranges between 6-69% [13]. Anxiety state was reported in 16-84% of celiac disease patients [14-16]. There are no studies in Jordan on the prevalence of psychological comorbidities in celiac disease. Therefore, the main purpose of our study was to determine the prevalence of anxiety and depressive symptoms among a sample of patients with celiac disease in Jordan.

## Materials and methods

This was a cross-sectional study in which we contacted adult patients (aged ≥18 years old) who had been diagnosed with celiac disease based on a patient-reported positive serological test and biopsy confirmation through the Friends of Celiac Disease Patients Association. This association is a non-profit organization that was established in 2012 and includes celiac disease patients, their carers, and healthcare professionals. It has the largest number of celiac disease patients in Jordan who have been diagnosed in public hospitals, the private sector, and the Royal Medical Services.

A questionnaire was sent electronically to patients using Google Forms (Google, Mountain View, California) through WhatsApp in August 2021. A short description of the study information was sent in the WhatsApp message along with a link to the questionnaire and a tick box for the patient's consent. Patients who agreed to participate were asked to provide their consent by ticking the box before scrolling through multiple-choice questions.

Questionnaires

The questionnaire consisted of three sections: the first one contained demographic data such as age, gender, marital status, education level, weight, height, presence of comorbidities, and family history of CD, in addition to disease-specific questions like duration since diagnosis, compliance with GFD and presence of symptoms. The second section contained the Arabic version of the Generalized Anxiety Disorder-7 (GAD-7) score, and it consisted of seven questions that evaluated anxiety symptoms. The third section contained nine questions that assessed depressive symptoms using the Arabic version of Patient Health Questionnaire-9 (PHQ-9). The Arabic versions of GAD-7 and PHQ-9 questionnaires have been previously validated in a Saudi study [17].

Measurements

The GAD-7 questionnaire that was used to assess anxiety consisted of seven questions, each graded from zero to three with a total score range between 0-21. Anxiety symptoms were considered present when the total score was five or more. The severity of anxiety symptoms was considered mild if the total score was five to nine, moderate if they scored 10-14, and severe if the total score exceeded 14.

Depressive symptoms were assessed using the PHQ-9 questionnaire that consisted of nine questions, each graded from zero to three with a total score ranging from 0-27. Depressive symptoms were considered present if the total score was five or more. The severity of depressive symptoms was considered mild if the score was five to nine, moderate if the score was 10-14, moderately severe if the score was 15-19, and severe if the score was ≥20.

Ethical considerations

This study was performed in accordance with the National Statement on Ethical Conduct in Human Research 2007 and the Declaration of Helsinki. Consent was obtained electronically from all patients who agreed to participate.

Statistical analysis

We used the Statistical Package for Social Sciences version 26 for Windows (IBM Inc., Armonk, New York) for the statistical analysis. Categorical variables were described using frequencies and percentages. Mean and standard deviation were calculated for the continuous variables. Spearman correlation was used to assess the relationship between anxiety symptoms, depressive symptoms, and the categorical variables. A p-value <0.05 was considered statistically significant.

## Results

A total of 133 patients responded and filled out the questionnaires. The mean age of the patients was 33.9 ± 11.22 years, and the majority (82.7%) were females. Of the patients, 71.4% had a diploma or higher education level, around half of the patients (53.4%) were married, and only 35.3% of the patients had a job. About 57.1% of the patients had their disease diagnosed one to five years prior to the study. Nearly a third of the patients (31.6%) were not compliant with a GFD, and more than half of the patients (56.4%) were symptomatic at the time of the questionnaire. Around 42.9% of the patients had comorbidities; the most common were thyroid disease and diabetes mellitus. The patients' characteristics are shown in Table 1.

**Table 1:** Celiac disease patient characteristics (n=133)

Variables	Mean ± SD, N (%)
Age (year)	33.92 ± 11.22
Weight (kg)	63.93 ± 16.86
Height (cm)	161.77 ± 8.10
Body mass index (kg/m^2)	25.71 ± 15.79
Anxiety score	10.13 ± 5.71
Depression score	11.86 ± 6.82
Sex	
Female	110 (82.7)
Male	23 (17.3)
Education	
Less than high school	7 (5.3)
High school	31 (23.3)
Diploma	26 (19.5)
Bachelor	56 (42.1)
Masters and PhD	13 (9.8)
Marital status	
Single	54 (40.6)
Married	71 (53.4)
Divorced	3 (2.3)
Widow	5 (3.8)
Body mass index categories	
Underweight	14 (10.5)
Normal weight	66 (49.6)
Overweight	36 (27.1)
Obese	17 (12.8)
Family history of celiac	
Yes	27 (20.3)
No	106 (79.7)
Employment status	
Employed	47 (35.3)
Unemployed	86 (64.7)
On a gluten-free diet	
Yes	108 (81.2)
No	25 (18.8)
Duration since diagnosis	
<1 year	12 (9.0)
1-5 years	76 (57.1)
6-10 years	24 (18.0)
11-15 years	12 (9.0)
16 years and more	9 (6.8)
Current symptoms of bloating, abdominal pain, diarrhea	
Yes	75 (56.4)
No	58 (43.6)
Comorbidities	
No	76 (57.1)
Yes	57 (42.9)

The mean depression score was 11.86 ± 6.82, and the mean anxiety score was 10.13 ± 5.71. The prevalence of anxiety and depressive symptoms were 85% and 82.7%, respectively.

The majority of patients with anxiety symptoms had mild levels of anxiety (39.1%), and only 15% had no or minimal anxiety symptoms (Table 2). Similarly, the most frequent depressive symptom level was mild depression (25.6%), and 17.3% had no or minimal depressive symptoms (Table 3).

**Table 2:** Levels of anxiety symptoms in celiac disease patients

Variable	Frequency	Percentage
No or minimal anxiety	20	15
Mild anxiety	52	39.1
Moderate anxiety	30	22.6
Severe anxiety	31	23.3
Total	133	100

**Table 3:** Levels of depressive symptoms in celiac disease patients

Variable	Frequency	Percentage
No or minimal depression	23	17.3
Mild depression	34	25.6
Moderate depression	32	24.1
Moderately severe depression	21	15.8
Severe depression	23	17.3
Total	133	100

There was no significant correlation between the presence of symptoms, duration since diagnosis, being on a GFD, or any of the demographic variables and the presence of anxiety or depressive symptoms.

## Discussion

Our study showed that anxiety and depressive symptoms are frequent among CD patients, which is consistent with previous studies [11,13-16]. We did not find any correlation between anxiety and/or depression and being on GFD, the presence of symptoms, female gender, and the time since diagnosis. This corresponds with the results of two studies by Fera et al. and Häuser et al., with the exception of female gender, which the latter found to be associated with higher rates of anxiety [15,16].

The prevalence of anxiety and depressive symptoms were 85% and 82.7%, respectively, which is higher than what was reported in the general Jordanian population (23.7% and 13.3%, respectively) [18,19]. The prevalence of anxiety symptoms in celiac patients in our study was similar to that reported in a German study by Häuser et al. which included 441 CD patients who were compared to inflammatory bowel disease patients and healthy controls which concluded that 84.8% of celiac patients were considered to have anxiety [16]. Our anxiety rate was higher than what was reported in the only study from the Middle East that we could find, in which Rostami-Nejad et al. reported a 67.8% anxiety rate in 283 celiac patients [20].

We found very high rates of depressive symptoms that far exceeded the reported prevalence in the literature, which ranged between 6-69% [13]. The high numbers that we found could be explained by different methods used to assess depressive and anxiety symptoms and the lack of public awareness of celiac disease. Additionally, the psychosocial consequences of following a GFD include the restriction of a patient's socialization with others, particularly in Jordan, where food is an essential part of social activities. Moreover, there is a financial burden in buying gluten-free products in a country with worsening poverty and rising unemployment rates, as well as a lack of governmental support for celiac patients. The impact of the COVID-19 pandemic may have also affected the results, as the study was done 18 months through the pandemic.

The pathophysiology of anxiety and depression in celiac disease is complex, with many theories to explain the association. These include the psychosocial effect of being diagnosed with celiac disease and the restrictive nature of GFD [21]; the role of low levels of tryptophan- an essential amino acid that is important for the synthesis of serotonin [22]; possible autoimmune reaction related to the release of immunogenic peptides that cross the blood-brain barrier and affect brain function [23,24]; vitamin deficiencies including vitamins B6 and B12, vitamin D, and folic acid [25-27]; cerebral hypoperfusion [28]; and gut microbiota which were found to be altered in celiac patients and were shown to have an effect on behavior and mood [29,30].

Our study had limitations. Firstly, the sample size was small. Secondly, selection bias was possible as the patients were recruited through the Friends of Celiac Disease Association. However, there are no hospital-based patient registries in Jordan, and to our knowledge, a large number of celiac patients in Jordan joined the association. Thirdly, reliance on the patient's self-reporting regarding diagnosis and compliance with a gluten-free diet. Fourthly, as the questionnaires did not contain patient identifiers, it was impossible to confirm the information from patients' medical records. Further, the assessment of only current anxiety and depressive symptoms was done using online questionnaires and lacked official psychiatric evaluation. Nonetheless, it was the first study in Jordan on this topic and one of few in the Middle East to evaluate the prevalence of mood disorders in celiac disease patients. 

## Conclusions

We found a high prevalence of anxiety and depressive symptoms among Jordanian CD patients. Based on these findings, gastroenterologists should screen patients for mood disorders and refer those who screen positively for further psychiatric evaluation.
